# Nitrogen-containing bisphosphonate induces a newly discovered hematopoietic structure in the omentum of an anemic mouse model by stimulating G-CSF production

**DOI:** 10.1007/s00441-016-2525-4

**Published:** 2016-11-05

**Authors:** Hirotada Otsuka, Hideki Yagi, Yasuo Endo, Satoshi Soeta, Naoko Nonaka, Masanori Nakamura

**Affiliations:** 1 Department of Oral Anatomy and Developmental Biology, School of Dentistry, Showa University, 1-5-8 Hatanodai, Shinagawa-ku, Tokyo, 142-8555 Japan; 2 Department of Pharmaceutical, Faculty of Pharmacy, International University of Health and Welfare, 2600-1 Kitakanamaru, Otawara-shi, Tochigi 324-8501 Japan; 3 Division of Molecular Regulation, Graduate School of Dentistry, Tohoku University, 4-1 Seiryo-machi, Aoba-ku, Sendai, 980-8575 Japan; 4 Department of Veterinary Anatomy, Nippon Veterinary and Animal Science University, 1-7–1 Kyonan-cho, Musashino-shi, Tokyo, 180-8602 Japan

**Keywords:** Newly discovered hematopoietic structure, Extramedullary hematopoiesis, Nitrogen-containing bisphosphoanate, SDF-1, G-CSF

## Abstract

We previously reported that the injection of nitrogen-containing bisphosphonate (NBP) induced the site of erythropoiesis to shift from the bone marrow (BM) to the spleen. Our previous study established a severely anemic mouse model that was treated with a combination of NBP with phenylhydrazine (PHZ), which induced newly discovered hematopoietic organs in the omentum. No reports have shown that new hematopoietic organs form under any condition. We characterized the structures and factors related to the formation of these new organs. Splenectomized mice were treated with NBP to inhibit erythropoiesis in the BM and then injected with PHZ to induce hemolytic anemia. The mice showed severe anemia and wine-colored structures appeared in the omentum. Some hematopoietic cells, including megakaryocytes, and well-developed sinuses were observed in these structures. Numerous TER119-positive erythroblasts were located with cells positive for PCNA, a cell proliferation marker. C-kit-positive cells were detected and mRNAs related to hematopoiesis were expressed in these structures. Moreover, TER119-positive erythroblasts emerged and formed clusters and hematopoiesis-related factors were detected in the omentum of mice treated with NBP and PHZ. The levels of G-CSF in the serum and hematopoietic progenitor cells (HPCs) in the peripheral blood were increased upon treatment with both NBP and PHZ. These results suggest that the induced hematopoietic structures act as the sites of erythropoiesis and that NBP-induced G-CSF production causes HPC mobilization, homing and colonization in the omentum because they constitutively express some factors, including SDF-1; thus, the newly discovered hematopoietic structure in this study might be formed.

**Electronic supplementary material:**

The online version of this article (doi:10.1007/s00441-016-2525-4) contains supplementary material, which is available to authorized users.

## Introduction

In adult mammals, hematopoiesis occurs in the bone marrow (BM) under normal conditions. BM possesses a specialized microenvironment called the ‘niche’ that maintains hematopoietic stem cells (HSCs) (McGrath and Palis 2008; Palis 2009). The stem cell niche, which is composed of cellular compartments such as osteoblasts, endothelial cells and adipocytes, has been reported to support hematopoiesis, hematopoietic cell homing, the establishment of resistance for self-renewal and the differentiation of hematopoietic lineage cells (Johns and Christopher 2012; Rankin et al. 2012). The niches in the BM are classified into two main types: an osteoblastic niche and a vascular niche. Osteoblasts produce several cytokines, such as G-CSF, GM-CSF and SDF-1. The conditional ablation of osteoblasts results in a reduction in the number of hematopoietic cells, including HSCs (Visnjic et al. 2004; Johns and Christopher 2012). In contrast, the factors leading to the establishment of HSCs within the vascular niche are not well understood. It has been shown that the recruitment of progenitors, including megakaryocytes, to the vascular niche involves signaling through SDF-1 and FGF-4 (Avecilla et al. 2004). The vascular niche seems to be associated with extramedullary hematopoiesis in the liver and spleen through the expression of SDF-1, which plays an essential role in the homing of HSCs and hematopoietic progenitor cells (HPCs) to BM (Inra et al. 2015; Mendt and Cardier 2015).

In adult mice, erythropoiesis occurs in the BM and spleen throughout life, even under normal conditions. The spleen is the main site of extramedullary hematopoiesis in response to acute anemia to maintain hematopoietic homeostasis. The liver has also been reported to promote extramedullary erythropoiesis in response to anemia in splenectomized mice (Lenox et al. 2009). In certain pathological conditions, extramedullary erythropoiesis is also detected in several organs, including the liver, lungs and hemal nodes (Halder et al. 2003; Bowling et al. 2008; Lenox et al. 2009). It has been reported that several factors induce extramedullary erythropoiesis, bone marrow failure, accelerated erythropoiesis, such as hypoxia and a marked stimulation of granulopoiesis (O’Malley 2007). Hypoxia caused by severe anemia is a primary stimulus for myelostimulatory extramedullary hematopoiesis, whereby increased erythropoietin (EPO) production in the kidney stimulates the proliferation and maturation of erythroid precursors (Lacombe and Mayeux 1998; Paliege et al. 2010). In humans, therapeutic G-CSF and GM-CSF treatments infrequently cause extramedullary hematopoiesis in the spleen and in other embryonic hematopoietic sites (Lacombe and Mayeux 1998). Recently, G-CSF was found to mobilize HSCs and HPCs from the BM to peripheral tissues, thus expanding the extramedullary pool of stem cells available for extramedullary hematopoiesis (Greenbaum and Link 2011; Li et al. 2012).

Bisphosphonates (BPs) are potent inhibitors of osteoclast-mediated bone resorption and are used as therapeutic agents against bone resorptive disorders, such as osteoporosis and metastatic bone diseases (Body 2003; Cremers et al. 2003). Nitrogen-containing bisphosphonates (NBPs) have strong anti-bone resorptive effects that are much more powerful than non-nitrogen-containing bisphosphonates (non-NBPs) and they exhibit inflammatory side effects (Endo et al. 1999; Nakamura et al. 1999; Yamaguchi et al. 2010). Our previous study established extramedullary erythropoiesis in livers without anemia and changes in serum EPO concentrations following the injection of NBP in splenectomized mice (Otsuka et al. 2011). We also reported the establishment of a severely anemic mouse model following treatment with both NBP and phenylhydrazine (PHZ) to induce hemolytic anemia and identified a new hematopoietic structure in the omentum (Otsuka et al. 2016). This is the first report of the newly induced hematopoietic structure in mice, even in anemic conditions.

Here, we identified the morphological features and formation mechanism of the newly induced structure in this study. Our data provide insight into understanding the mechanism of initial extramedullary erythropoiesis.

## Materials and methods

### Animals

**Fig. 1:**
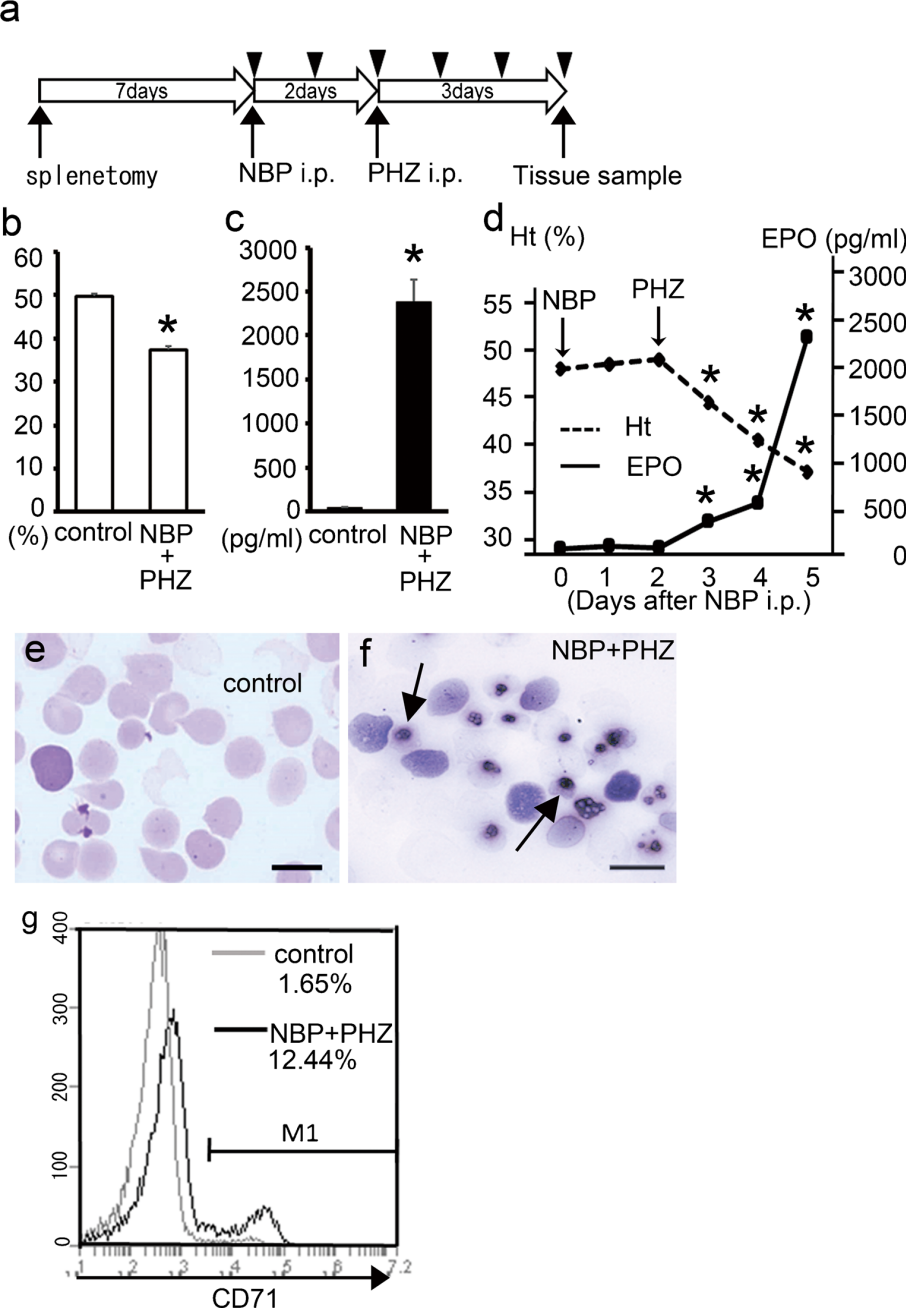
Experimental schedule and analysis of the peripheral blood. **a** A schematic of the experimental schedule. Blood samples were collected daily (*arrowhead*) and tissue samples were obtained 3 days after PHZ injection. **b** The hematocrit values at 3 days after PHZ (5 days after NBP) injection. A significant reduction was observed in the animals treated with both NBP and PHZ compared with the controls. **c** The serum EPO concentration at 3 days after PHZ (5 days after NBP) injection. A significant increase was observed in the animals treated with both NBP and PHZ. **d** The time-dependent change in the hematocrit values (*dashed line*) and serum EPO concentrations (*solid line*) in the animals treated with both NBP and PHZ. A significant reduction in the hematocrit values and an increase in the EPO concentrations were observed 1 day after PHZ injection. **e**, **f** Blood smears from control animals (**e**) and animals treated with both NBP and PHZ (at 3 days after PHZ; **f**) were stained with May-Grünwald Giemsa stain. Nucleated erythroid cells were easily detected in the mice treated with both NBP and PHZ (NBP + PHZ; *arrows*). **g** Flow cytometry analysis of CD71-positive erythroid lineage cells in the peripheral blood at 3 days after the PHZ (5 days after NBP) injection, which were gated as TER119-positive cells. The majority of cells in the controls were single-positive TER119 cells, whereas the number of TER119- and CD71-double-positive cells was significantly increased following treatment with both NBP and PHZ (NBP + PHZ). *Error bars* standard error of the mean (SEM). *Scale bars* (**e**, **f**) 10 μm. *P < 0.05 (vs. control or 0 day)

Sixty female BALB/c mice (6 weeks old) were obtained from Sankyo Laboratories (Tokyo, Japan) and housed under specific pathogen-free conditions. The mice were anesthetized with an intraperitoneal injection of sodium pentobarbital (50 mg/kg) and splenectomies were performed, as described in a previous report (Otsuka et al. 2011). The mice were divided into 2 groups, a splenectomized control group (20 mice) and a splenectomized and NBP and PHZ treatment group (40 mice). Seven days after splenectomy, NBP (40 μmol/kg) or sterile saline (control) was intraperitoneally injected into the splenectomized mice. Two days after NBP injection, the mice were treated with PHZ (50 mg/kg) or saline (control) and then euthanized 3 days after PHZ treatment. The experimental schedule is shown in Fig. 1a. The NBP used in this study was 4-amino-1-hydroxybutylidene-1, 1-bisphosphonate (AHBuBP), which was prepared as previously described (Endo et al. 1999; Nakamura et al. 1999).

All experimental protocols used in this study were reviewed and approved by the Animal Care Committee of Showa University (Permit Number: 15018, 16015).

### Blood analyses

Peripheral blood samples were collected and centrifuged at 10,000*g* for 5 min to determine hematocrit values and the sera were separated by centrifugation at 1,000*g* for 15 min. To measure the serum EPO and G-CSF levels, we used an EPO Mouse ELISA Kit and a G-CSF Mouse ELISA Kit (R & D Systems, Minneapolis, MN, USA), according to the manufacturer’s protocols and whole blood was prepared as a blood smear and stained using the May-Grünwald Giemsa staining technique.

### Antibodies and other materials

The monoclonal antibodies used in this study are listed below. The purified anti-mouse TER-119, Gr-1, B220, CD3 and PCNA and PE-conjugated anti-mouse TER-119 antibodies were purchased from BD Pharmingen (San Diego, CA, USA). APC-conjugated CD71, PE-conjugated anti-Sca1 and APC-conjugated anti-c-kit antibodies were purchased from Biolegend (San Diego, CA, USA). Biotinylated anti-c-kit antibody was purchased from Abcam (Cambridge, UK). Normal rabbit IgG, biotinylated goat anti-rat IgG, biotinylated goat anti-mouse IgG, biotinylated goat anti-hamster IgG, Texas Red-conjugated goat anti-Rat IgG antibodies and an avidin-biotin complex kit (ABC Elite standard kit) were purchased from Vector Laboratories (Burlingame, CA, USA). The anti-mouse F4/80 antibody was obtained from AbD Serotec (Kidington, UK). The Lineage Cell Depletion kit was purchased from Miltenyi Biotec (Bergisch Gladbach, Germany).

### Flow cytometry

To analyze the erythroid lineage cells in the peripheral blood, the cells were isolated from the peripheral blood, as previously described (Otsuka et al. 2016), washed using FACS solution (1 mM EDTA, 0.2 % BSA and 0.1 % NaN_3_ in PBS) and incubated with PE-conjugated anti-TER-119 and APC-conjugated anti-CD71 antibodies (1:200) or rat IgG (isotype control) in 1 % BSA in PBS.

To detect the hematopoietic precursor cells in the BM and peripheral blood, the cells from each tissue were obtained, depleted of erythrocytes, washed with FACS solution and incubated with a Lineage Cell Depletion kit according to the manufacturer’s protocol. These labeled cells were separated using AutoMACS Pro (Miltenyi Biotec) and the separated lineage-negative cells were stained with PE-conjugated anti-Sca1 and APC-conjugated anti-c-kit antibodies (dilute 1:200) or rat IgG (isotype control) in 1 % BSA in PBS.

After washing, the cells were resuspended in FACS solution and analyzed using a BD Accuri C6 Flow Cytometer (BD Bioscience, Rockville, MD, USA). The data were collected for 10,000 events and subsequently analyzed using the C6 Sampler software (BD Bioscience).

### Tissue preparation

The tissue samples were fixed in 4 % paraformaldehyde prepared in PBS, washed in 20 % sucrose-PBS, embedded in O.C.T. compound (Sakura Finetek Japan, Tokyo, Japan) and quickly frozen in a mixture of acetone and dry ice. Frozen sections (8 μm thick) were cut, placed on SILANE-coated glass slides and air-dried.

### Histology and immunohistochemistry

Some sections were stained with hematoxylin-eosin (HE). The remaining sections were rinsed in PBS and the sections for PCNA, a cell proliferation marker, were incubated in citrate buffer (pH 6.0) for 10 min at 121 °C for antigen retrieval. The sections were fixed in 1 % H_2_O_2_ in PBS for 30 min to quench endogenous peroxidases. After several PBS washes, the sections were incubated in 5 % normal goat serum in PBS. These sections were then incubated with anti-TER119, anti-Gr-1, anti-CD3, anti-B220, anti-F4/80 and anti-PCNA antibodies (1:100). After several rinses with PBS, the sections were incubated with biotinylated goat anti-rat, anti-mouse or anti-hamster antibodies, followed by a solution containing the avidin-biotin-horseradish peroxidase complex (ABC Elite standard kit). After washing, the sections were incubated with a mixture from the DAB detection kit (KPL, Gaithersburg, MD, USA). Hematoxylin was used as the counterstain.

### Immunofluorescence staining

The sections of newly discovered structures were rinsed in PBS and incubated with 5 % normal goat serum in PBS and then incubated with anti-c-kit monoclonal antibody (1:100). After several rinses with PBS, the sections were incubated with Texas Red-conjugated goat anti-rat IgG antibody. After the sections were washed with PBS, the sections were incubated with DAPI, washed with PBS and observed using a BZ-X710 fluorescence microscope (Keyence, Osaka, Japan).

### Transmission electron microscopy (TEM) and immunocytochemistry

The newly discovered hematopoietic structures were prepared for TEM. They were fixed in 2 % paraformaldehyde and 2.5 % glutaraldehyde in 0.1 M sodium cacodylate buffer, post-fixed in 2 % osmium tetroxide, dehydrated through a graded series of ethanol solutions, passed through propylene oxide and embedded in EPON 812 (TAAB, Aldermaston, UK).

For the immunocytochemical detection of TER-119-positive cells, the frozen sections were used in an immunohistochemical technique and re-fixed in 2.5 % glutaraldehyde before incubation with the DAB solution. After incubation with DAB, the sections were post-fixed in 2 % osmium tetroxide, dehydrated through a graded series of ethanol solutions and embedded in EPON 812.

Ultra-thin sections (80 nm thick) were cut and stained with uranyl acetate and lead citrate and observed under a Hitachi H-7600 transmission electron microscope (Hitachi, Tokyo, Japan).

### RT-PCR

Total RNA from the newly discovered hematopoietic structures and mesenteric lymph nodes of the NBP- and PHZ-treated mice and the omentum of the control and NBP- and PHZ-treated mice were isolated using an RNeasy Mini kit (QIAGEN, Hilden, Germany). The total RNA was reverse transcribed into cDNA using a PrimeScript RT reagent kit (Takara, Shiga, Japan). PCR was performed using a Veriti Thermal Cycler (Applied Biosystems, Foster City, CA, USA) with ExTaq (Takara) and the primer sequences and annealing temperatures are listed in Table 1.

**Table 1:** Sequence of used primer in this study

Name	Sequence	Anealing temperature (°C) Product size (bp)
*Scf*	F: 5′- AAGGAGATCTGCGGGAATCCTGTGA-3′ R: 5′- ACTGCTACTGCTGTCATTCCTAAGG-3′	58 525
*Il3*	F: 5′-CTGCCTACATCTGCGAATGA-3′ R: 5′-TTAGGAGAGACGGAGCCAGA-3′	58 161
*Csf1*	F: 5′-GACCCTCGAGTCAACAGAGC-3′ R: 5′-TGTCAGTCTCTGCCTGGATG-3′	58 236
*Csf2*	F: 5′-GGCCTTGGAAGCATGTAGAG-3′ R: 5′-CCGTAGACCCTGCTCGAATA-3′	58 161
*Csf3*	F: 5′-CTCAACTTTCTGCCCAGAGG-3′ R: 5′-TAGGTGGCACACAACTGCTC-3′	58 220
*Cxcl12*	F: 5′-CTTCATCCCCATTCTCCTCA-3′ R: 5′-GACTCTGCTCTGGTGGAAGG-3′	58 171
*Epor*	F: 5′-GGGATGGACTTCAACTACAG-3′ R: 5′-TGATGCGGTGATAGCGAGGAGA-3′	56 196
*Gata1*	F: 5′- ACCACTACAACACTCTGGCG -3′ R: 5′- CAAGAACTGAGTGGGGCGAT -3′	60 452
*Actb*	F: 5′-GCGTGACATTAAAGAGAAGCTG-3′ R: 5′-CTCAGGAGGAGCAATGATCTTG-3	60 376

### Colony forming assay

Colony-forming (CF) assays were performed according to the manufacturer’s protocol. The cells isolated from the BM and peripheral blood of the control mice and animals treated with NBP and PHZ (3 days after PHZ injection) were depleted of erythrocytes and the cells were plated in Methocult GF M3434 (1.3 % methylcellulose, 15 % fetal bovine serum, 2 % bovine serum albumin, 50 ng/ml SCF, 10 ng/ml IL-3, 10 ng/ml IL-6 and 5 IU/ml EPO) (STEMCELL Technologies, Vancouver, BC, Canada) and cultured at 37 °C for 10 days. Burst-forming unit-erythroid (BFU-E) and the other colonies (CFU-M, CFU-G, or CFU-GM) were counted after 10 days of culture.

### Statistical analysis

For the quantitative data analysis, *t* tests were used to determine the differences between paired samples. A *p *value of <0.05 was considered statistically significant.

## Results

### Blood analyses

We collected peripheral blood to evaluate the hematocrit values, measure the serum EPO concentrations and observe the erythrocytes in blood smears. In splenectomized mice treated with both NBP and PHZ (3 days after PHZ injection), a significant reduction in the hematocrit value was observed compared with the control mice (Fig. 1b). The concentration of EPO in the serum was also markedly increased at 3 days after PHZ injection (Fig. 1c). The hematocrit values in animals treated with both NBP and PHZ were significantly reduced in a time-dependent manner following NBP injection. The concentration of EPO in serum taken 1–3 days after PHZ treatment was also significantly increased compared with the serum taken 0 days after NBP injection (Fig. 1d).

The blood smears showed only normal enucleated erythrocytes in the control mice (Fig. 1e). In contrast, numerous nucleated erythrocytes and reticulocytes were observed in the peripheral blood of mice treated with both NBP and PHZ (Fig. 1f). Flow cytometry was performed to detect the proportion of CD71-positive cells in the TER119-positive peripheral blood cells. Most of the TER119-positive cells in the controls were CD71-negative erythrocytes, whereas the numbers of TER119- and CD71-double-positive cells were markedly increased in the animals treated with both NBP and PHZ (Fig. 1g).

These results indicate that splenectomized mice treated with NBP and PHZ become severely anemic with significantly increasing EPO levels to enhance erythropoiesis, similar to our previous report (Otsuka et al. 2016).

### Morphological study of the newly discovered structures

**Fig. 2:**
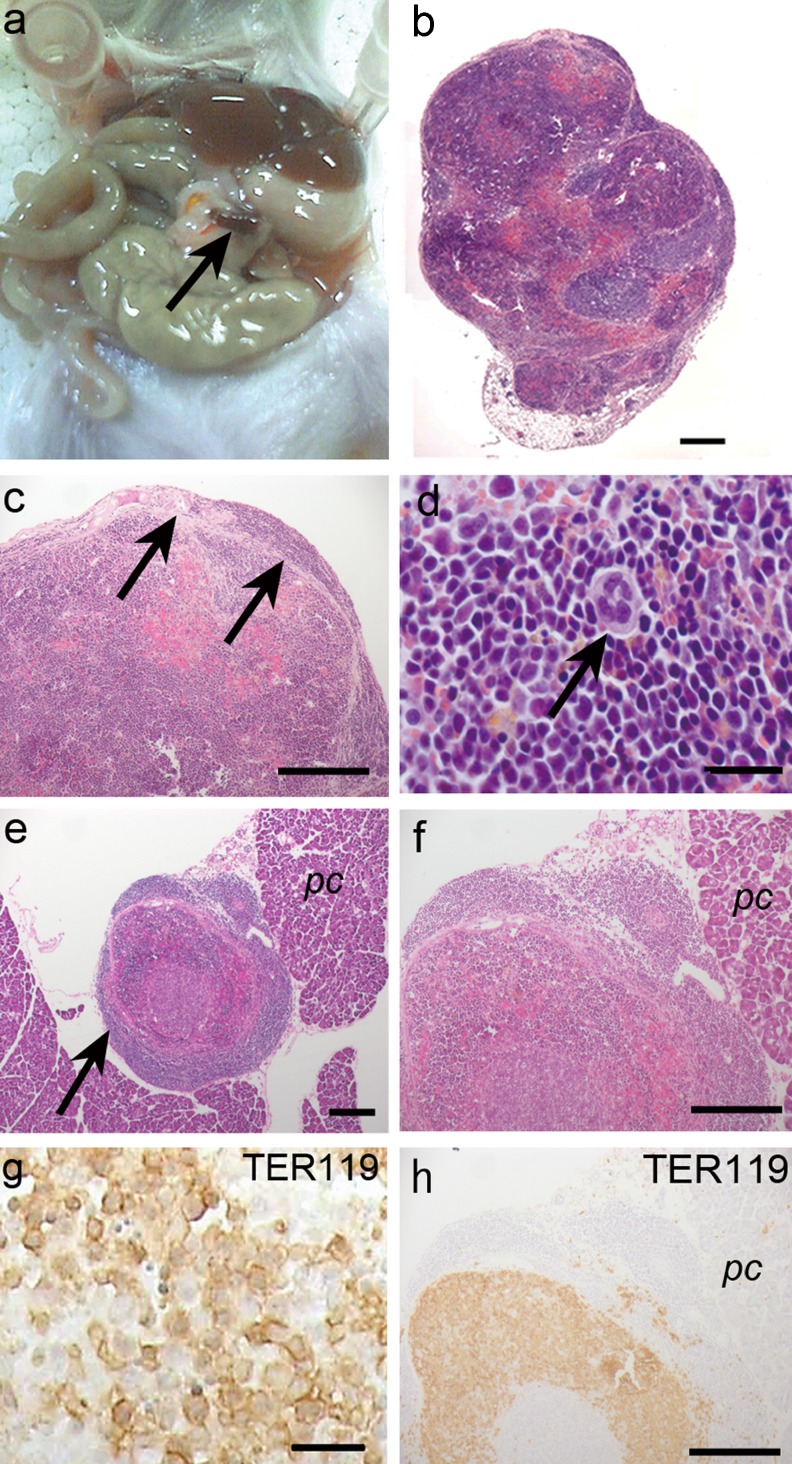
Morphological study of the newly induced structures in the abdominal cavity. **a** Macroscopic observation of the abdominal cavity. Wine-colored structures were observed in the omentum in splenectomized mice treated with both NBP and PHZ (*arrow*). **b** The histological analysis included HE staining of the newly identified structures. Some lymphoid follicle-like structures were observed. **c** High magnification of (**b**). A fibrous capsule surrounded the newly identified structure (*arrows*). **d** High magnification of (**c**). Numerous hematopoietic cells, including megakaryocytes (*arrow*), were observed in these structures. **e** HE staining of the newly identified structures (*arrow*) in the pancreas. **f** High magnification of (**e**). The fibrous capsule of the structure was not observed. **g**, **h** Immunohistochemical detection of the TER119-positive cells. Most of the cells in this structure were TER119-positive erythroblasts in the newly induced structures. *Scale bars* (**b**) 200 μm, (**c**, **e**, **f**, **h**) 100 μm, (**d**, **g**) 25 μm. *pc* pancreas

Wine-colored structures appeared in the omentum or pancreas of the animals treated with both NBP and PHZ (Fig. 2a). The size of these structures was 0.1–1.0 mm and they had fibrous capsules in the omentum (Fig. 2b, c), although the small size of the structures was not clearly distinct from the surrounding tissues (Fig. 2e, f). The histological study showed that there were lymphoid follicle-like cell clusters and well-developed sinusoids, similar to the spleen (Fig. 2b, c) and that some hematopoietic cells, including megakaryocytes, populated these structures (Fig. 2d). To detect whether erythropoiesis occurred in the induced structures, an immunohistochemical analysis was performed to detect the TER119-positive cells. Numerous TER119-positive, nucleated cells were detected in these structures (Fig. 2g, h).

These results indicate the possibility that newly discovered structures in a severe anemic mouse model play a role as the site of extramedullary hematopoiesis.

### Immunohistochemical detection and TEM observation of hematopoietic cells in the newly discovered structures

**Fig. 3:**
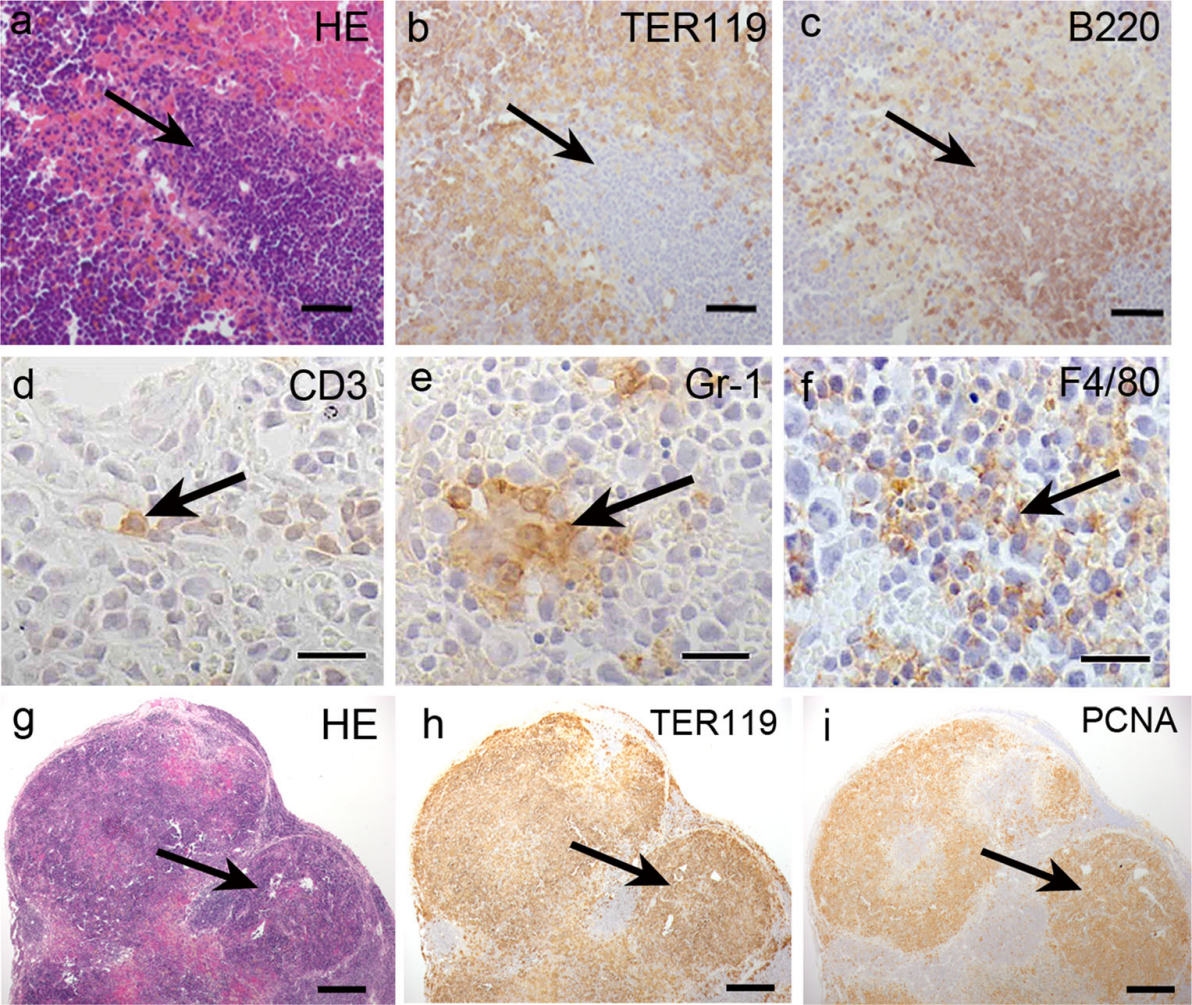
Histological study of the hematopoietic cells in the newly discovered hematopoietic structure. **a**–**c** The serial sections with HE staining (**a**) or immunohistochemistry for TER119 (**b**) and B220 (**c**). B220-positive cells formed a cluster (*arrow*) surrounded by a sinus and TER119-positive erythroblasts. There were no lymphoid follicles, such as those observed in lymph nodes. **d**–**f** Immunohistochemistry for CD3 (**d**), Gr-1 (**e**) and F4/80 (**f**). CD3-positive T-lymphocyte (*arrow*), Gr1-positive granulocytes (*arrow*) and F4/80-positive macrophages (*arrow*) were diffusely observed in these structures. **g** HE staining of the newly identified structures. **h** Immunohistochemical detection of the TER119-positive cells in serial sections of (**g**). Some lymphoid follicle-like structures were formed by TER119-positive erythroblasts (*arrow*). **i** Immunohistochemistry for PCNA in serial sections of (**h**). Most TER119-positive cells were located with PCNA-positive cells (*arrow*). *Scale bars* (**a**–**c**) 50 μm, (**d**–**f**) 20 μm, (**g**–**i**) 100 μm

To identify the hematopoietic cells in the newly discovered hematopoietic structures, we performed immunohistochemistry for the following lineage markers: Gr-1, B220, CD3 and F4/80. Nucleated cells formed clusters in these structures and these cells were TER119-negative and were surrounded by a sinus (Fig. 3a, b). These clusters were formed by B220-positive B lymphocytes (Fig. 3c). However, the B lymphocytes did not form lymphoid follicles. There were very few CD3-positive T-lymphocytes in these structures and they did not form a T lymphocyte region, as observed in the lymph node or spleen (Fig. 3d). Gr1-positive granulocytes existed in the periphery (Fig. 3e) and F4/80-positive macrophages were diffusely located in the region occupied by the TER119-positive erythroblasts in these structures (Fig. 3f). The lymphoid follicle-like clusters were filled with TER119-positive cells (Fig. 3g, h). Most of these cells were PCNA-positive proliferating cells (Fig. 3h, i).

**Fig. 4:**
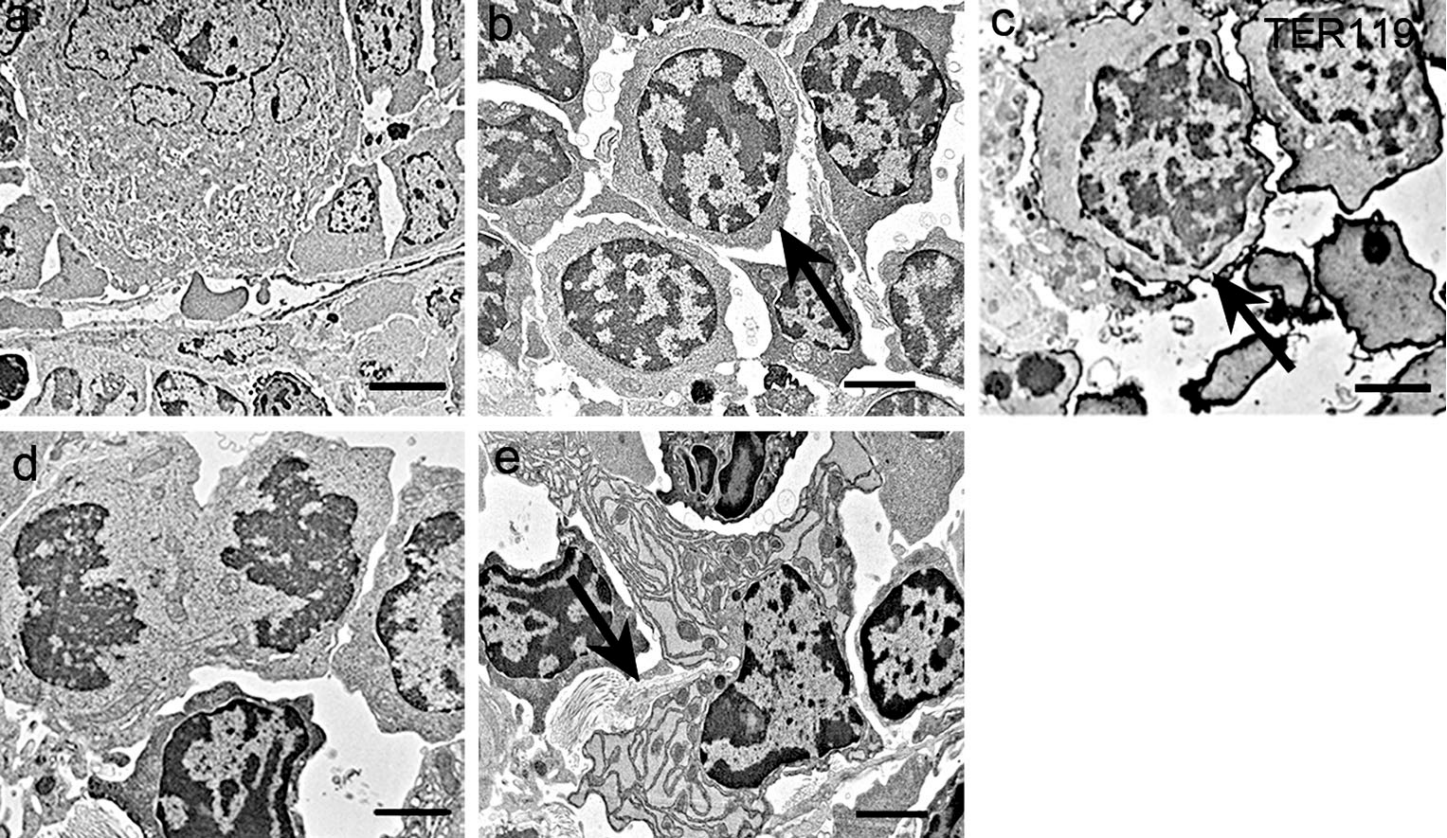
TEM observation of the newly discovered structure. **a** Some hematopoietic cells, including a megakaryocyte, were observed in this structure. **b** Some erythroblasts (*arrow*), which were filled with free ribosomes and thin cell organelles, were observed in the structures. **c** TER119-positive erythroblasts (*arrow*) and nucleated erythrocytes were present in the structures. **d** Cell division was observed in the structures. **e** A rough endoplasmic reticulum-rich fibroblast actively produced collagen fibers (*arrow*). *Scale bars* (**a**) 4 μm, (**b**) 2.5 μm, (**c**–**e**) 2 μm

The ultrastructural study indicated the presence of numerous hematopoietic cells, including megakaryocytes and erythroblasts, whose cytoplasm was filled with free ribosomes and thin cell organelles (Fig. 4a, b). Immunocytochemistry showed the presence of TER119-positive erythroblasts (Fig. 4c). Some of the cells were undergoing cell division (Fig. 4d) and some fibroblasts, which had abundant rough endoplasmic reticulum (rER), produced the collagen fibers (Fig. 4e).

These results indicate that active erythropoiesis occurs in these structures. The main function of the newly discovered structure is erythropoiesis and is not an acquired immunoreaction, such as that in lymphoid organs, because B cells did not form the lymphoid follicle and T cells did not form a specific region.

### Immunofluorescence staining of hematopoietic progenitor cells and mRNA expression of hematopoiesis-related factors in the newly discovered hematopoietic structures

**Fig. 5:**
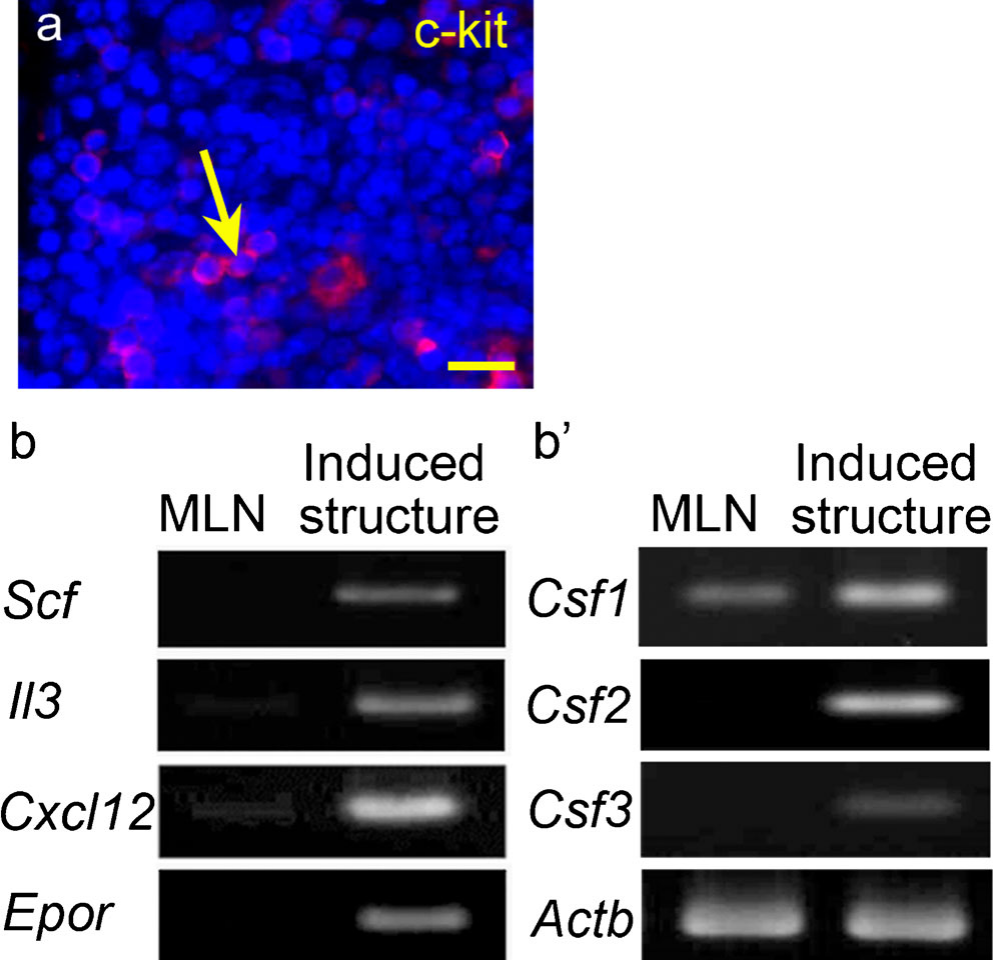
Immunofluorescence staining and RT-PCR analysis of the newly discovered hematopoietic structures. **a** Immunofluorescence staining of c-kit in the induced structure. Some c-kit-positive cells were observed in the structure. **b**, **b’** Comparison of the mesenteric lymph node (*MLN*) and the newly discovered hematopoietic structure (induced structure). Some mRNAs of cytokines (*Scf, Il3, Csf13* and *Cxcl12*) related to hematopoiesis and *Epor* were expressed in the newly discovered hematopoietic structure. *Scale bar* (**a**) 20 μm

We performed immunofluorescence staining for c-kit to identify the hematopoietic progenitor cells (HPCs) in the newly discovered structures. Some c-kit-positive cells were detected in these structures (Fig. 5a). Moreover, we performed RT-PCR of hematopoiesis-related cytokines to identify the microenvironment in the induced structures compared with the mesenteric lymph node. mRNAs associated with hematopoiesis, such as SCF (*Scf*), IL-3 (*Il3*), M-CSF (*Csf1*), G-CSF (*Csf2*), and GM-CSF (*Csf3*) and the homing factor SDF-1 (*Cxcl12*) were detected in the induced structures (Fig. 5b, b’). The erythropoiesis-related factors EPO receptor (*Epor*) were also detected in the newly discovered hematopoietic structures but not in the MLN (Fig. 5b, b’). The expression of β-actin (*Actb*) was used as an internal control. These results suggest that HPCs exist in the newly discovered hematopoietic structure and that the microenvironment in the structure is suitable for differentiation of HPCs and erythropoiesis.

### Histological analysis of the omentum

**Fig. 6:**
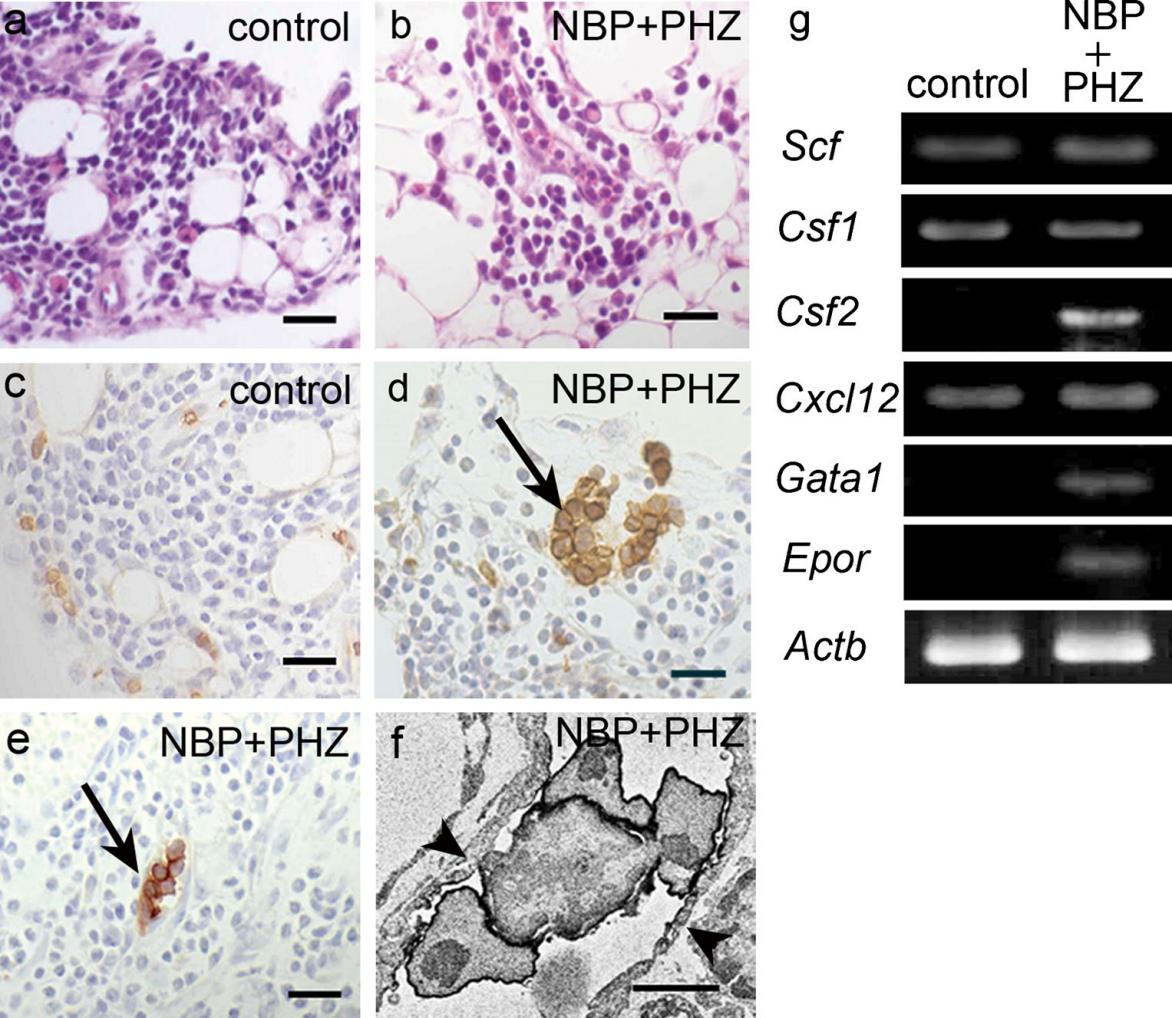
Histological study and RT-PCR analysis of the omental milky spot. **a**, **b** In the HE staining, no changes were observed in the NBP- and PHZ-treated mice (**b**) compared with the control mice (**a**). **c**, **d** Immunohistochemistry for TER119. TER119-positive cells were only present in the capillaries in the controls (**c**) but TER119-positive, nucleated cells formed a cluster in the animals treated with both NBP and PHZ (**d**, *arrow*). **e** Immunohistochemistry for CD71. CD71-positive erythroblast clusters were observed in the omentum of mice treated with both NBP and PHZ (*arrow*). **f** Immunocytochemistry for TER119. TER119-positive erythroblasts and nucleated erythrocytes accumulated in the sinusoidal lumen in the omentum of mice treated with NBP and PHZ. The *arrowhead* indicates the endothelium. **g** RT-PCR analysis of the omenta of the controls and animals treated with both NBP and PHZ. Some hematopoiesis-related factors were detected following treatment with NBP and PHZ. In addition, some mRNAs (*Scf, Csf1 and Cxcl12*) were also detected in the control animals. *Scale bars* (**a**–**e**) 25 μm, (**f**) 4 μm

The newly discovered hematopoietic structures usually emerged in the omentum. Therefore, we performed morphological observations in the omentum to identify the progenitor structure for hematopoiesis. We observed cell clusters called the omental milky spot in the omentum, which was composed of lymphocytes and macrophages around blood vessels and the lymphoid cells that had infiltrated between the adipocytes. A histological analysis of the HE staining indicated that there was no change in the omental milky spot following treatment with both NBP and PHZ compared with the control (Fig. 6a, b). Based on immunohistochemistry, the TER119-positive erythrocytes were only observed in the blood vessels in the control (Fig. 6c), whereas the TER119- and CD71-positive, nucleated cells formed clusters in the omentum of mice treated with both NBP and PHZ (Fig. 6d, e). The TER119-positive, nucleated erythroblasts and erythrocytes formed an agglomeration in the capillary lumen (Fig. 6f). These results show that the omental milky spots are not likely affected by hypoxia but that the mobilized erythroid progenitor cells colonize in the omentum following treatment with both NBP and PHZ.

### mRNA expression of hematopoiesis-related factors in the omentum

We performed RT-PCR to identify the omentum in the control animals and animals treated with both NBP and PHZ to identify the expression of cytokines related to hematopoiesis. SCF (*Scf*), MCSF (*Csf1*) and SDF-1 (*Cxcl12*) were expressed in the omentum of both groups but GATA1 (*Gata1*) and EPO receptor (*Epor*), which are related to erythropoiesis, were only detected in the animals treated with both NBP and PHZ (Fig. 6g). These results suggest that erythropoiesis is induced in the omentum following treatment with both NBP and PHZ. The omentum seems to possess a suitable microenvironment for hematopoiesis because it expresses several factors, including SDF-1, even in normal conditions (Fig. 6g). Increased levels of these cytokines might support the observed mobilization of HPCs homing, colonization and differentiation in peripheral tissues in this study.

### CF assays and quantitative analyses of HPCs in BM and peripheral blood

**Fig. 7:**
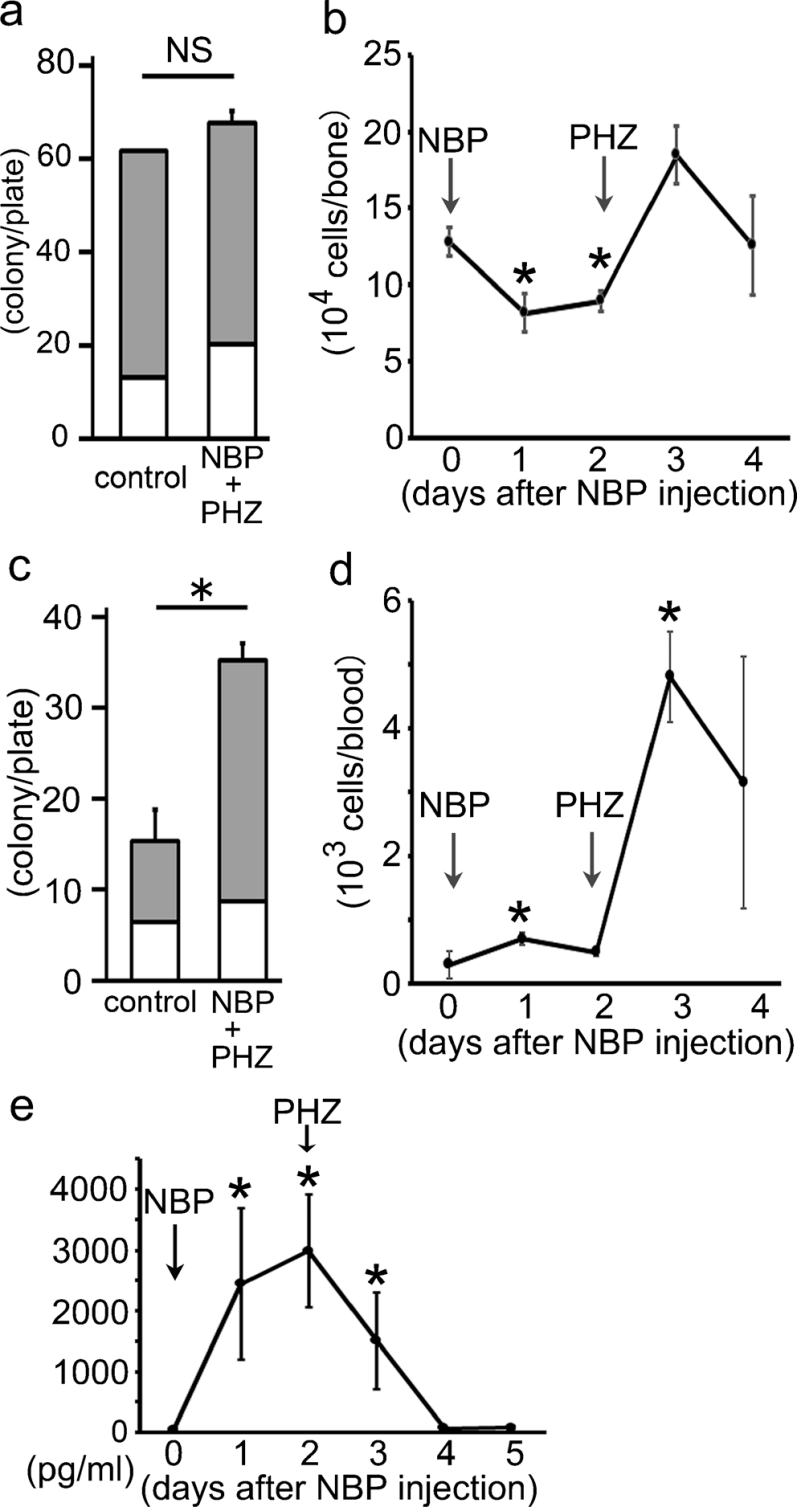
Hematopoietic progenitor cells in the BM and peripheral blood. **a** Colony formation (CF) assay in the BM. No significant change was observed between the controls and animals treated with both NBP and PHZ (*NHP + PHZ)*. **b** Number of lineage-negative, c-kit-positive HPCs (LK cells) in the BM. The number of these LK cells was significantly decreased at 1 and 2 days after NBP injection. **c** CF assay in peripheral blood. Colony formation was markedly increased in the peripheral blood of animals treated with both NBP and PHZ compared with the controls. **d** The number of LK cells in the peripheral blood. The number of LK cells was significantly increased at 1 day after NBP injection. **e** The time-dependent change of serum G-CSF concentrations. The serum G-CSF concentration was significantly increased at 1–3 days after NBP injection compared with 0 days after NBP injection. *NS* no statistical significance. *P < 0.05 (vs. control or 0 day)

To evaluate the hematopoietic ability of mononuclear cells in the BM and peripheral blood, we performed a CF assay. In the BM, no significant change was detected between the control mice and mice treated with both NBP and PHZ (Fig. 7a). However, a significant increase in the number of colonies was induced in the cells from the peripheral blood of mice treated with both NBP and PHZ compared with the control mice (Fig. 7c). We also analyzed the number of lineage-negative and c-kit-positive HPCs (LK cells) in the BM and peripheral blood after NBP injection to detect the mobilization of the HPCs from the BM to the peripheral tissues. The population of LK cells was significantly decreased in the BM at 1–2 days after NBP injection, whereas it was markedly increased in the peripheral blood during the same period following treatment (Fig. 7b, d). Moreover, these LK cells entirely recovered to their normal levels in the BM at 3 days after NBP injection (Fig. 7b). However, the number of cells was still significantly increased in the peripheral blood after PHZ injection (Fig. 7d).

### Measurement of G-CSF in the serum

We next measured the serum G-SCF concentrations because G-CSF is the key cytokine associated with the mobilization of HSCs and the transport of HPCs from the BM to peripheral tissues. One day after NBP injection, the G-CSF levels increased precipitously and remained at a high concentration for up to 3 days after NBP treatment, which was well correlated with the decrease in the number of LK cells in the BM (Figs. 7b, e; S1). NBP has been reported to enhance granulopoiesis and inflammation (Nakamura et al. 1999), which might be mediated by G-CSF stimulation. Our results suggested that G-CSF production was stimulated by NBP treatment, which then provoked the mobilization of HPCs from the BM to the peripheral tissue.

## Discussion

In adult vertebrates, the main hematopoietic organ normally becomes localized in the BM. However, extramedullary hematopoiesis occurs under several conditions (Halder et al. 2003; Bowling et al. 2008; Lenox et al. 2009). Many reports have shown that extramedullary erythropoiesis in the liver was induced in splenectomized mice under acute anemic conditions (Halder et al. 2003; Lenox et al. 2009). Our previous study also showed that a severely anemic mouse model was established by treating the animals with both NBP and PHZ and that numerous erythroblasts formed clusters in the liver (Otsuka et al. 2016). We unexpectedly observed an undiscovered, wine-colored structure in the abdominal cavity in this model (Otsuka et al. 2016). In the present study, we showed the morphological features, functions and the mechanism of the formation of this newly discovered structure. These newly discovered structures always emerged in the omentum or pancreas and were filled with numerous erythroblasts. Well-developed sinuses were also detected. These newly discovered structures are very similar to the hemal node and/or hemolymph node (Turner 1969; Casteleyn et al. 2008; Zidan and Pabst 2010). Hemal nodes or hemolymph nodes are lymphoid organs. The hemal node has some lymphoid follicles, similar to lymph nodes but the sinusoidal systems are more extensive, similar to the spleen and contain blood instead of lymph, producing a red macroscopic appearance (Zhang et al. 2013). These are observed in a wide range of animals, including man. However, these organs have never been identified in the mouse (Van den Broeck et al. 2006). These structures provide the site of hematopoiesis under certain pathological conditions, such as acute anemia (Cerutti and Guerrero 2008) but their main roles are to destroy erythrocytes and mediate an immune response by trapping reticulo-endothelial components and processing antigenic material and erythrocytes (Schultz et al. 1973). The newly discovered hematopoietic structures in this study were the site of extramedullary erythropoiesis but they did not play a function as lymphoid organs. In addition, the newly discovered hematopoietic structures did not form lymphoid follicles and T cell regions. These results indicated that the newly induced hematopoietic structure was not a lymphoid organ.

We observed the newly discovered hematopoietic structures in the omentum with and without fibrous capsule. The omentum is composed of two mesothelium sheets enclosing a loose connective tissue that contains some hematopoietic cells, macrophages, lymphocytes and dendritic cells, called the “omental milky spot” (Platell et al. 2000). Omental milky spots directly contact with adipocytes, lacking fibrous capsule. The small size of the structures induced in this study was not clearly distinct from surrounding tissue, although the larger structures formed fibrous capsules. In addition, rER-rich fibroblasts, actively producing collagen fibers, were observed in the larger structures. By these results, we believe that the simple accumulation of hematopoietic cells in the early stage of the formation process developed into an independent organ and a fibrous capsule on a larger structure is formed to maintain the structure such as lymph nodes ontogeny (Bailey and Weiss 1975).

New Zealand Black mice display an age-associated progression to chronic lymphocytic leukemia and have well-developed omental milky spots and a dysfunctional spleen, which are involved in extramedullary hematopoiesis (Takemori et al. 1995; Takemori 2007). There is another report that the consecutive injection of a high dose of EPO induces erythropoiesis in the omental milky spots in the ddY strain (Hirai et al. 1994). The omental milky spot contains many macrophage precursors and forms a microenvironment that promotes differentiation into macrophages (Zhu et al. 1997). The fetal mouse omentum has been shown to be a source of precursors that exclusively reconstitute Ly1+ B cells (Kantor et al. 1992). The transposition of the omental pedicle to injured organs has been used to promote healing and the omental transposition experiment reported the expansion of the mass of milky spots and increased VEGF expression (Litbarg et al. 2007). The peritoneum, including the omentum, is known to provide a hypoxic microenvironment that is a suitable niche for cancer stem cells and is associated with peritoneal metastasis (Miao et al. 2014). Moreover, the HSCs are localized to the hypoxic region of the BM (Nombela-Arrieta et al. 2013; Morikawa and Takubo 2015; Kocabas et al. 2015). These reports support that the microenvironment of the omentum is similar to the niche for hematopoiesis. We also detected some cytokines that are related to hematopoiesis, including SCF and SDF-1, in the omentum, even in normal conditions. In particular, SDF-1 plays an essential role in the homing of HSCs and HPCs and is associated with extramedullary hematopoiesis (Inra et al. 2015; Mendt and Cardier 2015). The features of the omentum identified in this study indicated that it is a suitable microenvironment for hematopoiesis because the omentum is more likely to be associated with the formation of the newly discovered structure. Interestingly, we only observed the hematopoietic structure in the omentum, not in the mesenterium; these peritoneal tissues have different origins: the dorsal mesogastrium and dorsal mesenterium, respectively (Robinson 1899). The cytokines identified in the omentum were not detected in the mesenteric lymph nodes. Therefore, the microenvironments in these two peritoneal tissues might be different.

G-CSF is a cytokine related to granulopoiesis and G-SCF production is enhanced in inflammation and stimulates granulopoiesis. G-CSF has other functions, namely to suppress osteoblasts lineage cells and inhibit the expression of some factors, SDF-1, SCF and VCAM-1, which results in the mobilization of HSCs and HPCs from the BM to peripheral tissues (Semerad et al. 2005; Greenbaum and Link 2011). In this study, we detected an increase in both the serum G-SCF levels and the numbers of lineage-negative and c-kit-positive HPCs in the peripheral blood following NBP treatment. The relationship between bisphosphonates and G-CSF production is unclear but NBP injection induces some side effects, including granulopoiesis, inflammation, extramedullary hematopoiesis and necrosis (Nakamura et al. 1999; Yamaguchi et al. 2010) and mediates the significant release of IL-6, TNF-α and IL-1β (Endo et al. 1992). Moreover, fat-associated lymphoid clusters such as omental milky spots are induced by inflammation (Benezech et al. 2015). These results suggest that NBP injection induces G-CSF production and that G-CSF should play an essential role in the formation of the newly discovered hematopoietic structure.

Taken together, these results suggest that the increased G-CSF production following the NBP injection mediated HSCs and/or HPCs mobilization, which colonized the omentum and provided the niche for hematopoiesis by expressing some hematopoiesis-related factors, including SDF-1. Moreover, the high EPO level stimulated erythropoiesis, resulting in the formation of the new hematopoietic structures. Our study reveals a previously unknown mechanism of NBP, the function of the omentum and the relationship between the peritoneal adipose tissue and hematopoietic cells.

## Electronic supplementary material

Below is the link to the electronic supplementary material.

Fig. S1 (PDF 59 kb)
