# Supplementary material for: Nitrogen-containing bisphosphonate induces a newly discovered hematopoietic structure in the omentum of an anemic mouse model by stimulating G-CSF production
Source: Cell Tissue Res. 2016 Nov 5;367(2):297–309. doi: 10.1007/s00441-016-2525-4 (PMC5269465; doi:10.1007/s00441-016-2525-4)
Supplement: Supplementary file 1 — (PDF 59 kb) [file 441_2016_2525_MOESM1_ESM.pdf]

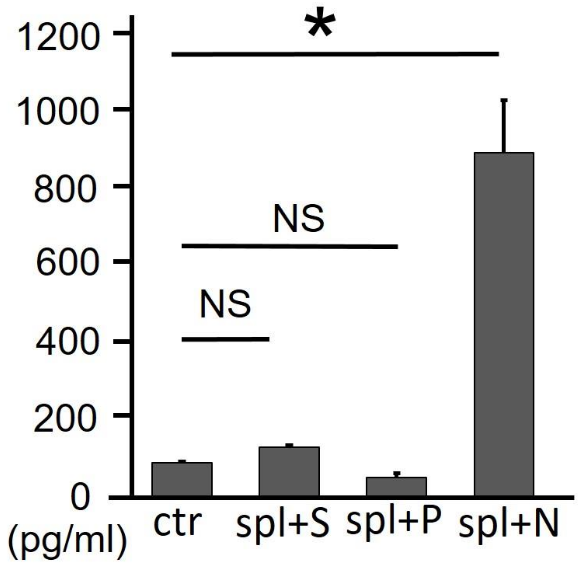

Figure S1

Serum G-CSF concentrations of each group. The mice were divided into 4 groups: a non-treatment group (control, crt), a splenectomized and saline injection group (spl+S), a splenectomized and PHZ treatment group (PHZ alone, spl+H), a splenectomized and NBP treatment group (NBP alone, spl+N). Serum was collected 3 days after treatment.

The serum G-CSF concentration NBP-only group (spl+N) was significantly increased compared with the control group (ctr). Asterisks indicate statistical significance (P<0.05), and NS denotes no significance versus the control. The error bars indicate the standard error of the mean (SEM).
